# Supplementary material for: Chloride intracellular channel (CLIC) proteins function as fusogens
Source: Nat Commun. 2024 Mar 7;15:2085. doi: 10.1038/s41467-024-46301-z (PMC10920813; doi:10.1038/s41467-024-46301-z)
Supplement: Supplementary file 1 — Supplementary Information [file 41467_2024_46301_MOESM1_ESM.pdf]

Supplementary information

Chloride intracellular channel (CLIC) proteins function as fusogens

Bar Manori[1,*], Alisa Vaknin[2,*], Pavla Vaňková[3], Anat Nitzan[4], Ronen Zaidel-Bar[4], Petr Man[3], Moshe Giladi[1,5,#], Yoni Haitin[1,6,#]

[1] Department of Physiology and Pharmacology, Faculty of Medicine, Tel-Aviv University, Tel-Aviv, 6997801, Israel

[2] School of Chemistry, Raymond & Beverly Sackler Faculty of Exact Sciences, Tel Aviv University, 6997801 Tel Aviv, Israel

[3] Institute of Microbiology of the Czech Academy of Sciences, Division BioCeV, Prumyslova 595, 252 50 Vestec, Czech Republic

[4] Department of Cell and Developmental Biology, Faculty of Medicine, Tel-Aviv University, Tel-Aviv, 6997801, Israel

[5] Tel Aviv Sourasky Medical Center, Tel Aviv, 6423906, Israel

[6] Sagol School of Neuroscience, Tel Aviv University, Tel Aviv, 6997801, Israel

[*] These authors contributed equally

[#] Correspondence: moshegil@post.tau.ac.il or yhaitin@tauex.tau.ac.il

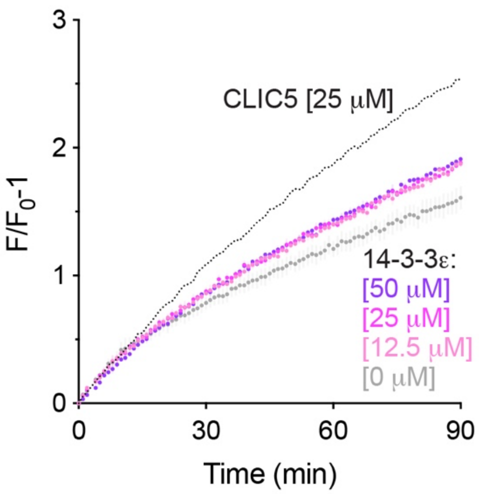

Supplementary Figure 1. 14-3-3ε does not induce lipid mixing. Dose-response analysis of liposomal membrane mixing by 14-3-3ε using the R18 fluorescence unquenching assay. CLIC5 (25 μM; Fig. 3a) is shown as a positive control. For all experiments, data are presented as mean ± SEM, n = 3 independent experiments.

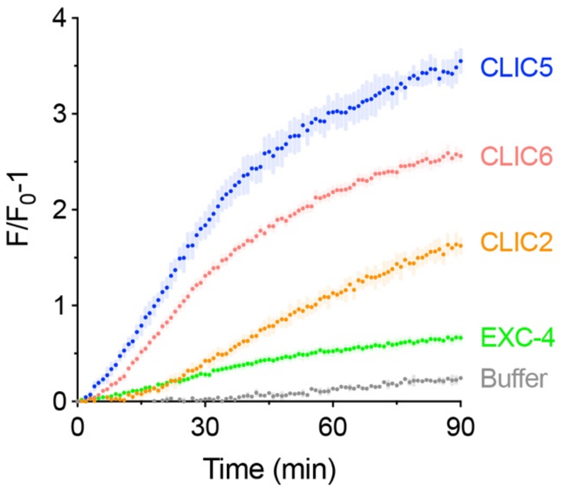

Supplementary Figure 2. CLIC paralogs induce membrane fusion. Analysis of liposomal membrane mixing by the indicated CLIC paralogs (25 μM) using the R18 fluorescence unquenching assay. For all experiments, data are presented as mean ± SEM, n = 2-6 independent experiments.

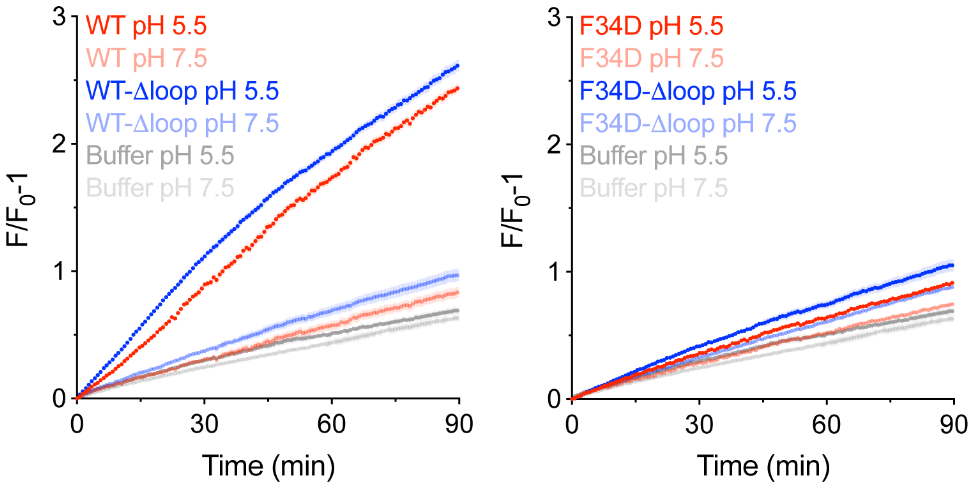

Supplementary Figure 3. Functional analysis of CLIC5-Δloop. Analysis of liposomal membrane mixing by the indicated CLIC constructs (25 μM) using the R18 fluorescence unquenching assay. For all experiments, data are presented as mean ± SEM, n = 6 independent experiments.

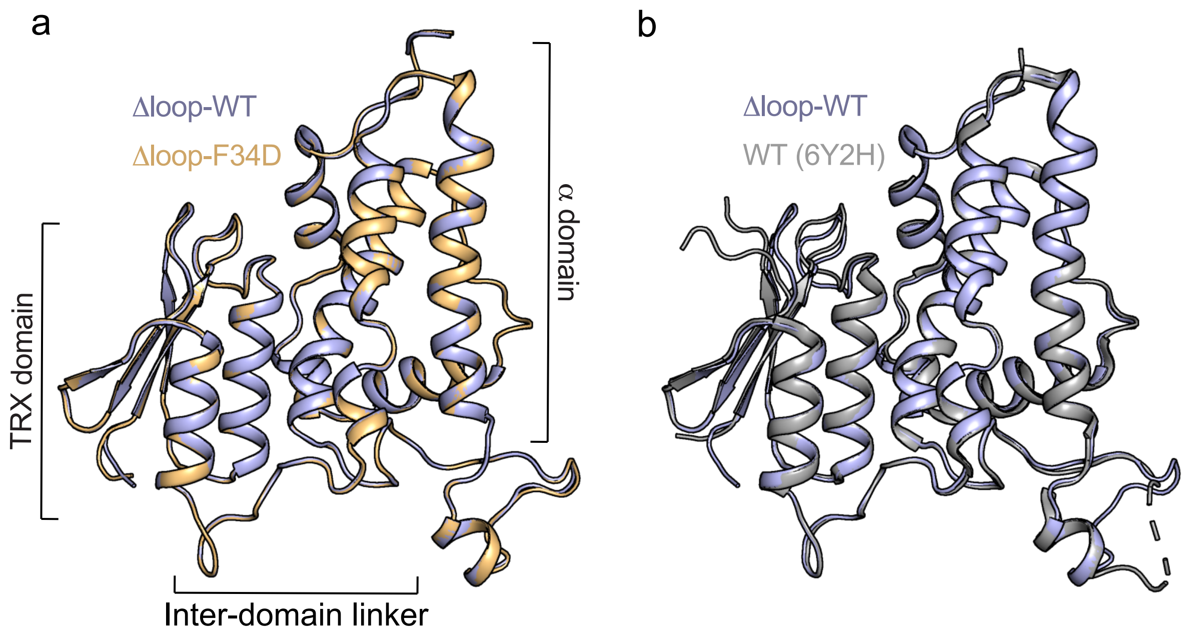

Supplementary Figure 4. CLIC5-F34D does not induce significant structural perturbations. (a, b) Superposition of CLIC5-Δloop-WT (purple) and CLIC5-Δloop-F34D (orange) (a) or CLIC5-WT (PDB 6Y2H) (grey) (b). Neither the mutation nor the deletion of the loop results in major structural alterations.

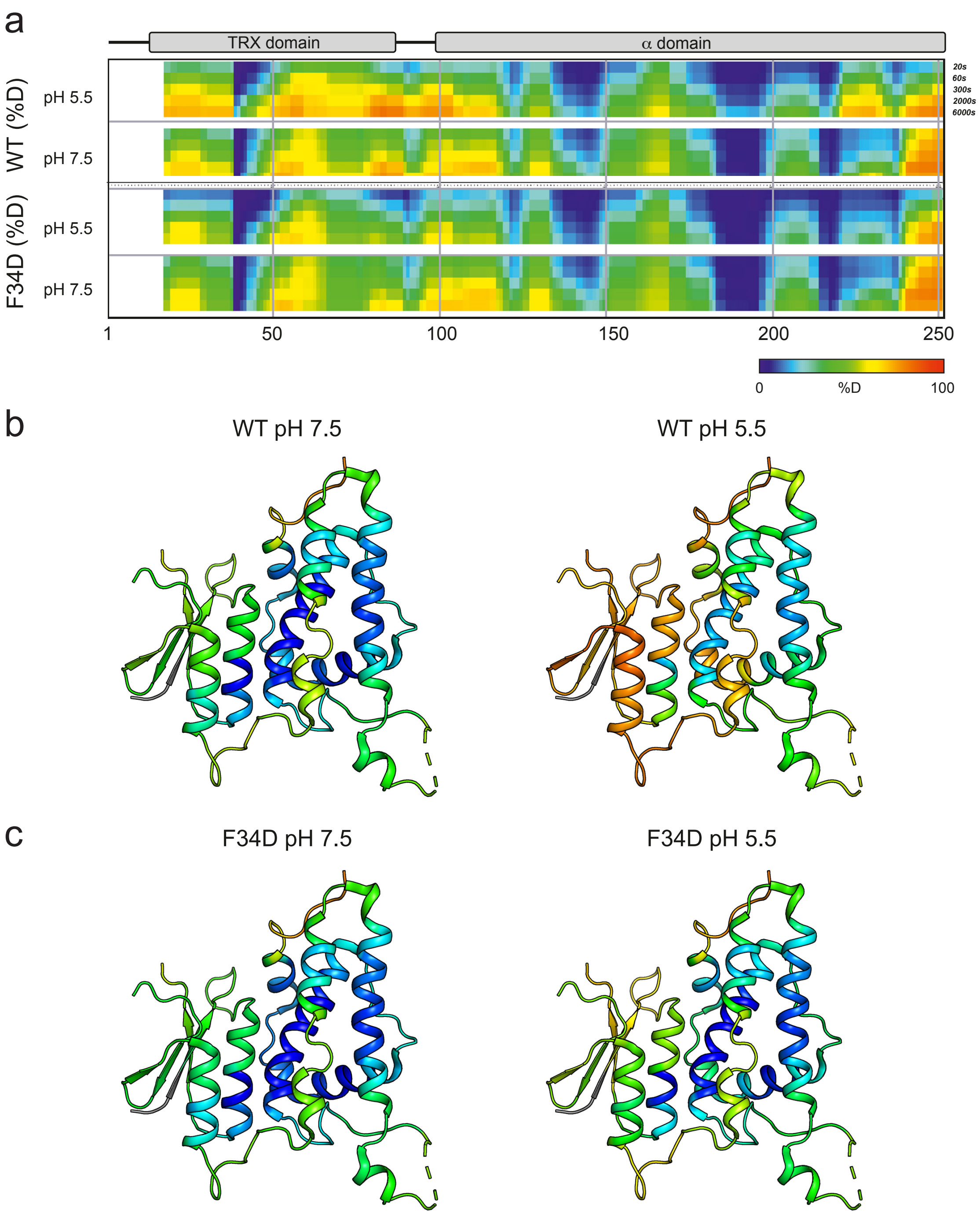

Supplementary Figure 5. Deuterium uptake profiles of CLIC5-WT and CLIC5-F34D. (a) Deuteration levels at the indicated time points for CLIC5-WT (upper panels) and CLIC5-F34D (lower panels), at the indicated pH values. **(b, c)** The deuterium uptake levels at 60 seconds (pH 7.5) or 6000 seconds (pH 5.5) are projected onto the structure of CLIC5-WT (PDB 6Y2H), as indicated.

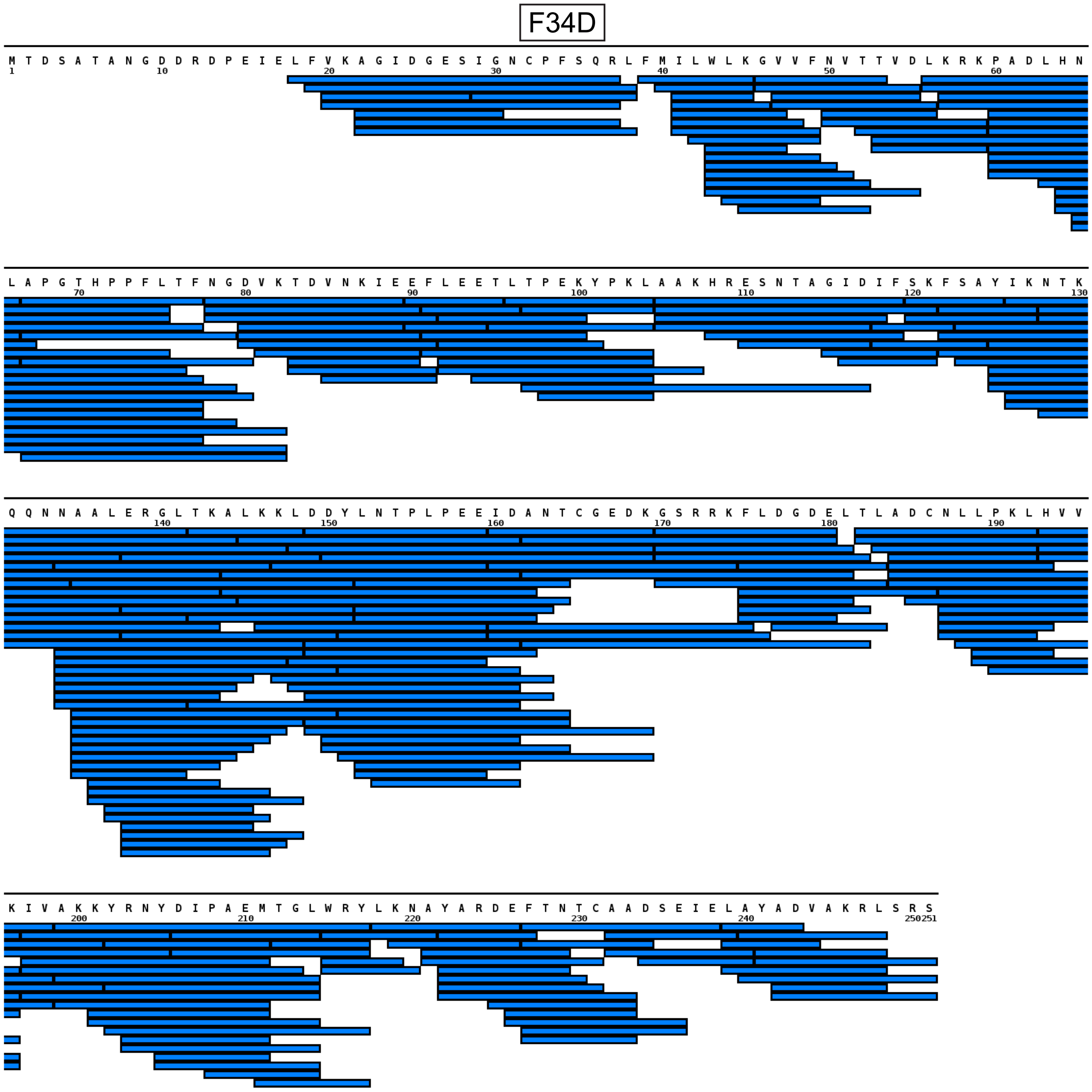

Supplementary Figure 6. Sequence coverage in HDX-MS experiments. Sequence coverage in HDX-MS experiments. The map shows peptides generated after online digestion using immobilized pepsin/nepenthesin-2 under HDX-MS conditions.

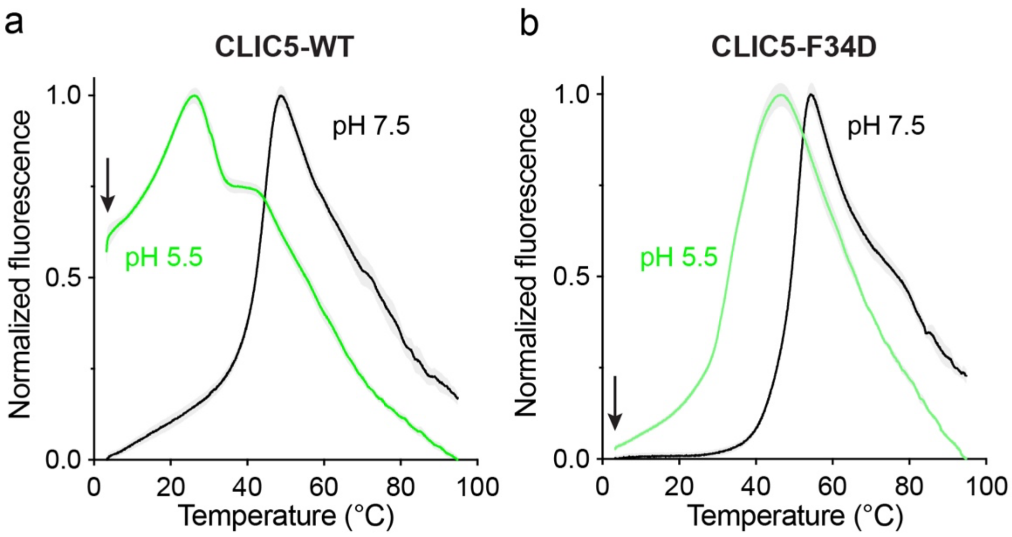

Supplementary Figure 7. TSA analysis of CLIC5-WT and CLIC5-F34D. (a, b) Normalized SYPRO Orange fluorescence-temperature relation of CLIC5-WT (a) and CLIC5-F34D (b) at the indicated pH values. Basal fluorescence signals are indicated (arrows). For all experiments, data are presented as mean ± SEM, n = 6 independent experiments.

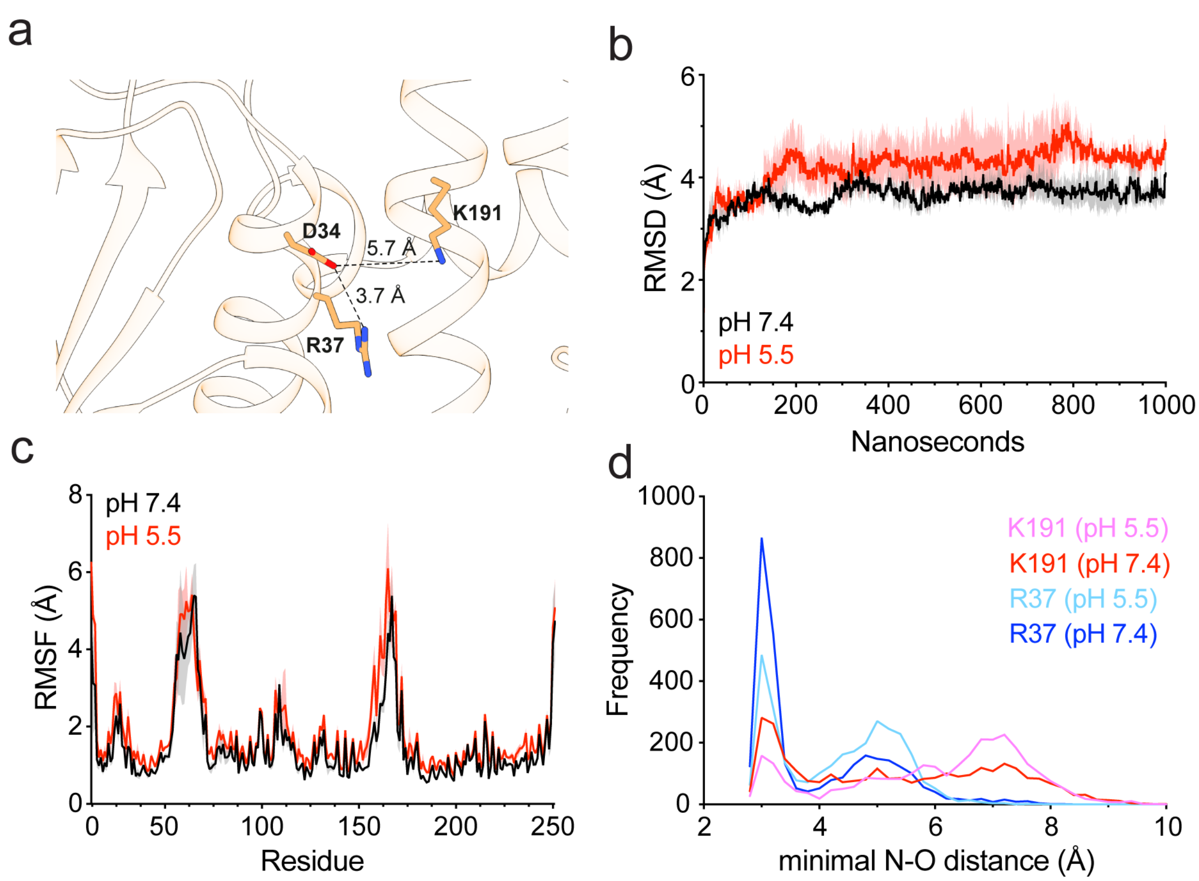

Supplementary Figure 8. Molecular dynamics simulations of CLIC5-F34D. (a) A zoomed view on the interdomain interface. D34, R37, and K191 are shown as sticks. The minimal side-chains N-O distances are shown as dashed lines. **(b)** Average root mean square deviation (RMSD) of CLIC5-F34D along the simulation trajectories performed at the indicated pH values (n = 3 for each condition). The plateau indicates that the simulations reached convergence. The shaded area indicates the standard deviation. **(c)** Average root mean square fluctuation (RMSF), representing the per-residue spatial fluctuation relative to their average position along the simulation trajectories, at the indicated pH values. **(d)** Distance distributions of the minimal N-O distance between the carboxylate of D34 and the side chains of R37 or K191 at the indicated pH values. The peak at ~3 Å is consistent with salt-bridge formation.

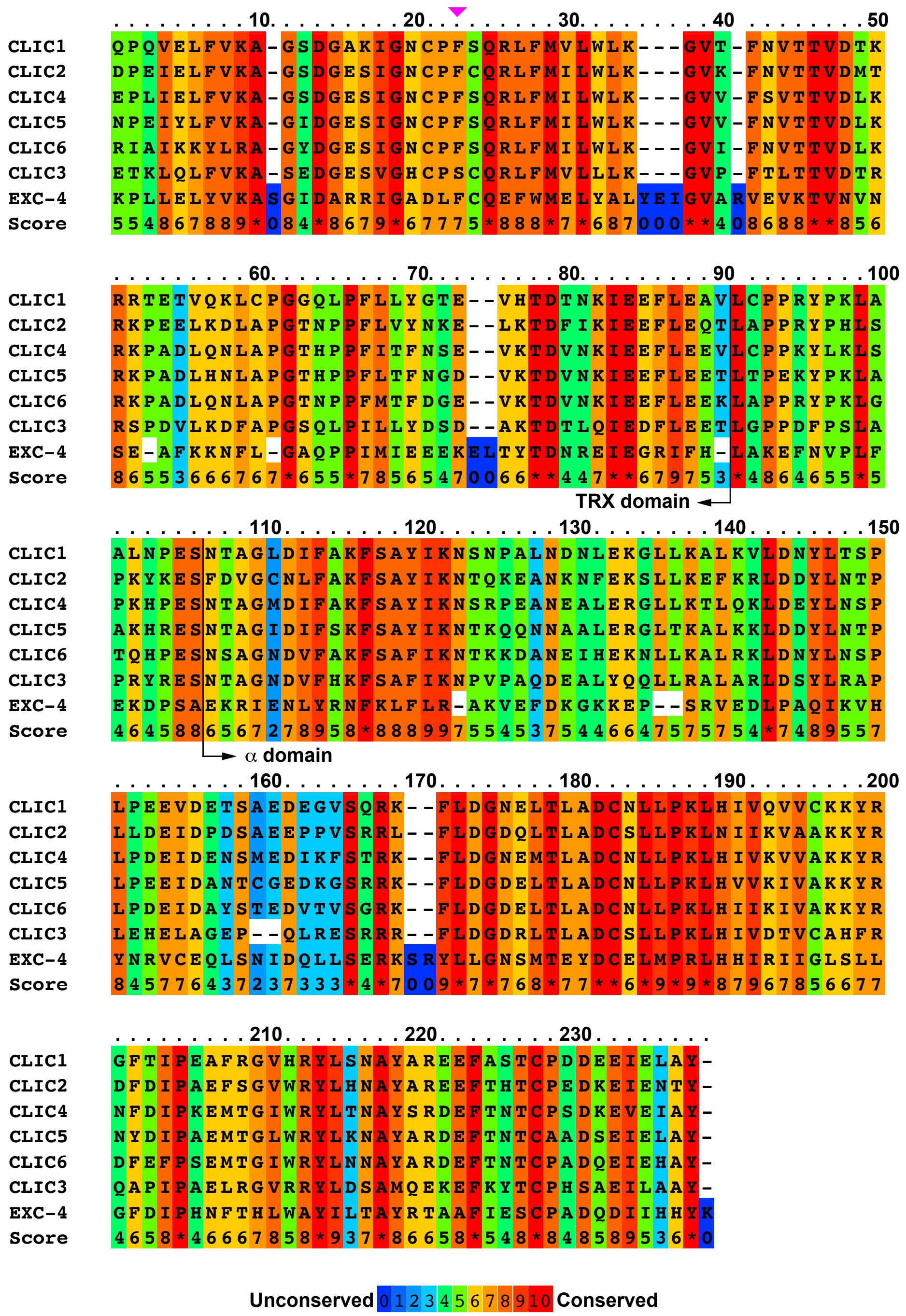

Supplementary Figure 9. CLIC proteins sequence similarity. CLIC domain PRALINE Multiple sequence alignment (https://www.ibi.vu.nl/programs/pralinewww/) of all human CLIC family members and EXC-4. Residues are shown in green and grey, respectively. TRX and α domain boundaries and CLIC5-F34 are indicated (black arrows and purple arrowhead, respectively).

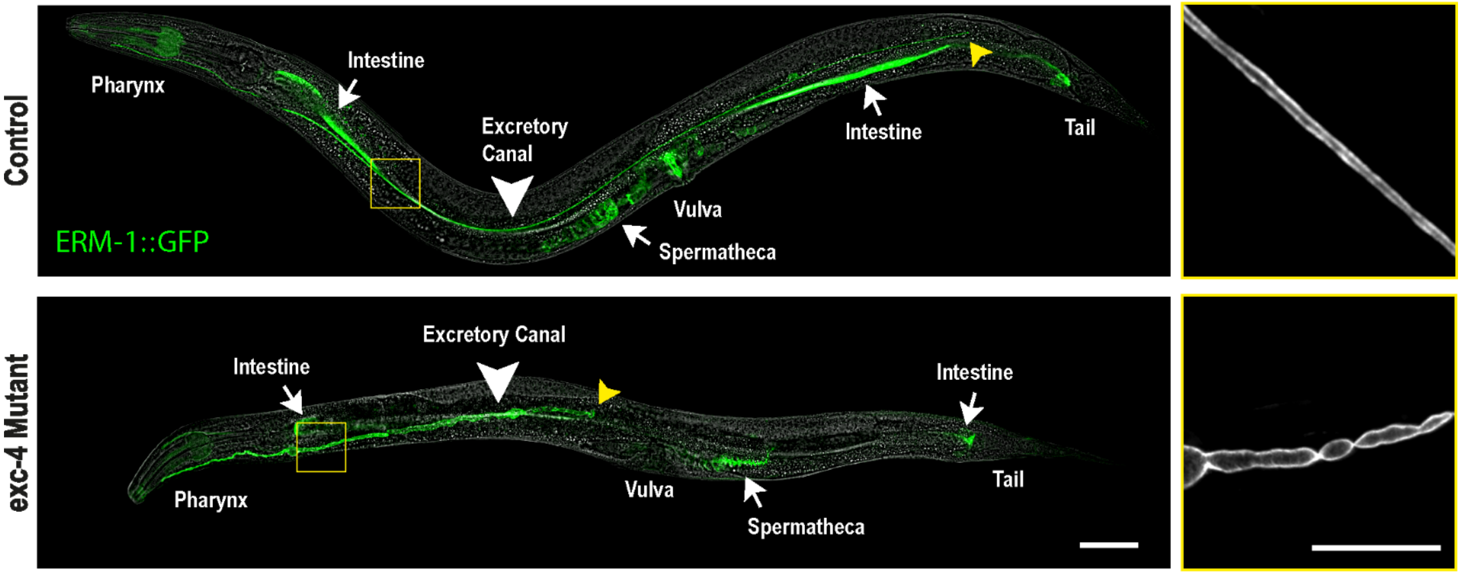

Supplementary Figure 10. ERM-1 localization in the excretory canal of control and *exc-4* F38D mutant *C. elegans*. Representative images of L4 stage worms expressing ERM-1::GFP (green). Control worm (upper panel) with an excretory canal extending from the pharynx to the tail, and *exc-4* mutant worm (lower panel) with an excretory canal ending before the vulva and harboring a distended lumen and visible cysts. Yellow arrows indicate the end of the canal in each worm. ERM-1::GFP is expressed in the excretory canal (white arrowheads), where it localizes along the lumen in both control and *exc-4* F38D mutant worms. ERM-1 is also expressed in the intestine and spermatheca, which are marked with white arrows. Yellow rectangles mark the regions magnified on the right. Bars = 50 µm in whole worm view and 20 µm in enlargement.

Supplementary Table 1. Molecular dynamics simulations system setup.

	**WT**	**F34D**
**Simulation box dimensions (Å)**	81.2 x 86.6 x 58.8	80.9 x 84.6 x 58.1
**No. of atoms**	38,102	37,089
**No. of water molecules**	11,410	11,076
**Salt concentration (KCl)**	0.15 M	0.15 M
