# Supplementary material for: Chloride intracellular channel (CLIC) proteins function as fusogens
Source: Nat Commun. 2024 Mar 7;15:2085. doi: 10.1038/s41467-024-46301-z (PMC10920813; doi:10.1038/s41467-024-46301-z)
Supplement: Supplementary file 3 — Reporting Summary [file 41467_2024_46301_MOESM3_ESM.pdf]

nature portfolio

Corresponding author(s): Yoni Haitin and Moshe Giladi

Last updated by author(s): Feb 8, 2024

Reporting Summary

Nature Portfolio wishes to improve the reproducibility of the work that we publish. This form provides structure for consistency and transparency in reporting. For further information on Nature Portfolio policies, see our Editorial Policies and the Editorial Policy Checklist.

Statistics

For all statistical analyses, confirm that the following items are present in the figure legend, table legend, main text, or Methods section.

n/a	Confirmed	
☐	☒	The exact sample size (n) for each experimental group/condition, given as a discrete number and unit of measurement
☐	☒	A statement on whether measurements were taken from distinct samples or whether the same sample was measured repeatedly
☐	☒	The statistical test(s) used AND whether they are one- or two-sided<br>*Only common tests should be described solely by name; describe more complex techniques in the Methods section.*
☒	☐	A description of all covariates tested
☒	☐	A description of any assumptions or corrections, such as tests of normality and adjustment for multiple comparisons
☐	☒	A full description of the statistical parameters including central tendency (e.g. means) or other basic estimates (e.g. regression coefficient) AND variation (e.g. standard deviation) or associated estimates of uncertainty (e.g. confidence intervals)
☐	☒	For null hypothesis testing, the test statistic (e.g. F, t, r) with confidence intervals, effect sizes, degrees of freedom and P value noted<br>*Give P values as exact values whenever suitable.*
☒	☐	For Bayesian analysis, information on the choice of priors and Markov chain Monte Carlo settings
☒	☐	For hierarchical and complex designs, identification of the appropriate level for tests and full reporting of outcomes
☒	☐	Estimates of effect sizes (e.g. Cohen's d, Pearson's r), indicating how they were calculated

Our web collection on statistics for biologists contains articles on many of the points above.

Software and code

Policy information about availability of computer code

Data collection	Spectra Manager Suite 2.15.01 (Jasco),Chronos software (AxelSemrau), MetaMorph version 7.10.2.240 (Molecular Devices).
Data analysis	Spectra Manager Suite 2.15.01 (Jasco), Prism 10 10.0.3 (217), UCSF chimera 1.14, PyMol 2.0, Bruker Daltonics data analysis 5.3, MASCOT search engine 2.7, DeuteEx, MSTools, XDS, Phaser.

For manuscripts utilizing custom algorithms or software that are central to the research but not yet described in published literature, software must be made available to editors and reviewers. We strongly encourage code deposition in a community repository (e.g. GitHub). See the Nature Portfolio guidelines for submitting code & software for further information.

Data

Policy information about availability of data

All manuscripts must include a data availability statement. This statement should provide the following information, where applicable:

- Accession codes, unique identifiers, or web links for publicly available datasets
- A description of any restrictions on data availability
- For clinical datasets or third party data, please ensure that the statement adheres to our policy

Data supporting the findings of this study are available from the corresponding authors upon request.

Research involving human participants, their data, or biological material

Policy information about studies with human participants or human data. See also policy information about sex, gender (identity/presentation), and sexual orientation and race, ethnicity and racism.

Reporting on sex and gender	N/A
Reporting on race, ethnicity, or other socially relevant groupings	N/A
Population characteristics	N/A
Recruitment	N/A
Ethics oversight	N/A

Note that full information on the approval of the study protocol must also be provided in the manuscript.

Field-specific reporting

Please select the one below that is the best fit for your research. If you are not sure, read the appropriate sections before making your selection.

☒ Life sciences ☐ Behavioural & social sciences ☐ Ecological, evolutionary & environmental sciences

For a reference copy of the document with all sections, see nature.com/documents/nr-reporting-summary-flat.pdf

Life sciences study design

All studies must disclose on these points even when the disclosure is negative.

Sample size	No statistical methods were used to predetermine sample sizes. Required experimental sample sizes were chosen according to common practice in protein biochemistry and microscopy and were sufficient to ensure reproducibility. Statistical analysis was limited to determine mean ± SEM.
Data exclusions	Data were not excluded.
Replication	Absorbance (A410), fluorescence spectroscopy (FRET), and liposomal fusion analyses (DLS, R18 fluorescence unquenching, carboxyfluorescein fluorescence unquenching) were recorded at least twice on different occasions from different preparations, and all replications were included in the analyses. For the HDX-MS experiments, the 25 °C experiment was performed at five time points (20 s, 60 s, 300 s, 2000 s and 6000 s intervals), with two points (20 s and 300 s) triplicated (n=3). All replications were successful and included in the presented data. For C. elegans microscopy, data from 40 and 82 control and mutant worms were collected and included in the analyses performed. All replications were successful and included in the presented data.
Randomization	Randomization was not performed in this study as samples were predefined and did not require allocation into different experimental groups. The groups were prepared independently and compared under controlled conditions.
Blinding	Blinding was not performed in this study as the results are quantitative in nature and do not require subjective interpretation.

Reporting for specific materials, systems and methods

We require information from authors about some types of materials, experimental systems and methods used in many studies. Here, indicate whether each material, system or method listed is relevant to your study. If you are not sure if a list item applies to your research, read the appropriate section before selecting a response.

Materials & experimental systems

n/a	Involved in the study	
☒	☐	Antibodies
☒	☐	Eukaryotic cell lines
☒	☐	Palaeontology and archaeology
☐	☒	Animals and other organisms
☒	☐	Clinical data
☒	☐	Dual use research of concern
☒	☐	Plants

Methods

n/a	Involved in the study	
☒	☐	ChIP-seq
☒	☐	Flow cytometry
☒	☐	MRI-based neuroimaging

Animals and other research organisms

Policy information about studies involving animals; ARRIVE guidelines recommended for reporting animal research, and Sex and Gender in Research

Laboratory animals	C. elegans strains used in this study: N2 – wild type; SYS1728 - mir-232p::mNeonGreen::PH::tbb-2 3'UTR + NeoR with lineage reporter (provided by Zhuo Du, Chinese Academy of Sciences); RZB452 - exc-4 (msn213[F80D]); mir-232p::mNeonGreen::PH::tbb-2 3'UTR + NeoR with lineage reporter. Worm microscopy data were obtained at the L4 stage.
Wild animals	No wild animals were used in this study.
Reporting on sex	N/A
Field-collected samples	No field-collected samples were used in this study.
Ethics oversight	This study did not require an ethical approval.

Note that full information on the approval of the study protocol must also be provided in the manuscript.

Plants

Seed stocks	N/A
Novel plant genotypes	N/A
Authentication	N/A
